# 242. Association Between Procalcitonin and Antimicrobial Usage in Patients with COVID-19

**DOI:** 10.1093/ofid/ofac492.320

**Published:** 2022-12-15

**Authors:** Mahmoud H Aly, Eris Cani, Lendelle Raymond, Cosmina Zeana

**Affiliations:** BronxCare Health System, Monmouth Junction, New Jersey; BronxCare Hospital Center, Bronx, New York; BronxCare Health System, Monmouth Junction, New Jersey; Bronxcare Health System, NYC, New York

## Abstract

**Background:**

Patients with COVID-19 disease often receive antibiotics to treat suspected bacterial coinfections. Procalcitonin (PCT) is a biomarker used for suspected bacterial infections. The objective of this study is to evaluate the association between PCT and the use of antimicrobials in COVID-19 patients.

**Methods:**

This was a retrospective, cohort study of adult patients admitted with confirmed COVID-19 from March 30, 2020 to March 30, 2021. Data collected included demographics, baseline inflammatory markers including initial PCT and C-reactive protein (CRP) values, past medical history, initiation of empiric antibiotics, mechanical ventilation, in-hospital mortality, days of antibiotic therapy, and length of hospital stay (LOS). Univariate analyses were utilized to assess for any significant differences in demographics based on predefined initial PCT groupings (< 0.25 ng/ml (group 1), 0.25-0.49 ng/ml (group 2), and ≥ 0.5 ng/ml (group 3)). Multivariate analyses were performed to evaluate for any differences between initial PCT values and in-hospital mortality, LOS, and days of antibiotic therapy.

**Results:**

Out of 149 patients, 61.7% had an initial PCT value < 0.25 ng/ml, 17.45% had an initial value of 0.25-0.49 ng/ml, and 20.8% had an initial value ≥ 0.5 ng/ml. A total of 145 patients (97%) received empiric antibiotics. Univariate analysis among the three groups displayed a difference in the initial CRP value, which was higher in groups 2 and 3 versus group 1 (p < 0.001). Regression analysis controlling for initial CRP value found that patients in groups 2 and 3 had a higher duration of antibiotic therapy compared to group 1 (12 and 11 versus 8 days) (p < 0.001) and a longer LOS (17 and 15 vs 12 days) (p = 0.009). More patients (34.6%) were mechanically ventilated in group 2 compared to group 1 (14.1%) and group 3 (22.6%) with a trend toward significance (p = 0.059). Multivariate analysis found no significant association between PCT levels and mortality. The rate of in-hospital mortality in patients receiving invasive ventilation was higher in groups 2 and 3 (78% and 86%, respectively) compared to group 1 (54%, p < 0.001).

**Conclusion:**

When controlling for CRP, an initial PCT value > 0.25 ng/ml was associated with increased days of antibiotic therapy and longer duration of hospital stay in COVID-19 patients.

**Disclosures:**

**All Authors**: No reported disclosures.

